# Nurse managers’ experiences regarding the use of key performance indicators in developing work plans

**DOI:** 10.4102/phcfm.v14i1.3556

**Published:** 2022-11-30

**Authors:** Thembelihle S.P. Ngxongo, Judith N. Mdima Masondo

**Affiliations:** 1 Department of Nursing, Faculty of Health Sciences, Durban University of Technology, Durban, South Africa

**Keywords:** clinic, key performance indicators, knowledge, KwaZulu-Natal, nurse managers, primary health care, target, work plans

## Abstract

**Background:**

Apart from monitoring and evaluation, key performance indicators (KPIs) are used for planning of quality healthcare services and are essential for ensuring that work plans are strategic. Nurse managers (NMs) are required to use reports on the KPIs from the District Health Barometer to prepare work plans.

**Aim:**

The aim of the study was to explore the experiences of NMs regarding the use of KPIs in developing work plans.

**Setting:**

The study was conducted in one of the four local municipalities of iLembe district in KwaZulu-Natal.

**Methods:**

A qualitative research design was used. Data were collected through semistructured interviews with 20 NMs from seven primary health care clinics between 16 November 2020 and 24 December 2020; data were then thematically analysed.

**Results:**

Nurse managers experienced challenges related to knowledge creation, knowledge sharing and knowledge application regarding the use of KPIs in developing work plans. Possible strategies which, according to NMs, could facilitate the use of KPIs in developing work plans included support in the management role, skills development and orientation, provision of resources and participative management.

**Conclusion:**

Relevant knowledge management practices, including knowledge sharing, are essential for knowledge creation so that individuals develop task knowledge to fulfil role expectations. In the case of this study, knowledge creation for NMs could facilitate their appropriate use of KPIs in developing work plans.

**Contribution:**

It is recommended that NMs receive ongoing training on, and be actively involved in, data management and that mentoring and support be made available for NMs to facilitate the use of KPIs in developing work plans.

## Introduction

The overarching aim of universal health coverage is for all people who need health services to be able to access and receive high-quality care. The World Health Organization defines quality of care as ‘the degree to which health services for populations increase the likelihood of desired health outcomes and are consistent with evidence-based professional knowledge’.^1^ Therefore, quality of care can be measured and continuously improved through the provision of evidence-based care that takes into consideration the needs and preferences of service users.^1^ Quality provision of care in healthcare clinics requires competent and motivated healthcare professionals, as well as information systems that enable reviews and audits to take place.^1^ Thus, a well-established District Health Information System through which data is reported annually in the District Health Barometer supports the monitoring and evaluation system for the primary health care (PHC) system in South Africa as a strategy to facilitate quality provision of healthcare services.^2^ Data inform decisions regarding the flow of patients and the diversity of their needs in predictive workforce modelling, identify cost drivers and inform the culture of workplace safety within a healthcare environment.^3^

The PHC clinics have several data sets, referred to as key performance indicators (KPIs), with some specific targets attached to them for monitoring and evaluation.^4^ In its publication of the Sustainable Development Goals, the 2030 Agenda for Sustainable Development provided the goals and target descriptions that, if implemented at a country level, would lead towards a sustainable future.^5^ However, the 2030 Agenda leaves it to countries to adopt the targets, with each government setting its own national targets guided by the global level of ambition but taking into account national circumstances. Within each country, targets for each KPI at each level of operation (provincial, district and PHC levels) are set such that they are aligned to each other because they are all towards achievement of national targets.^5^

The KPIs for PHC clinics are determined based on the key performance areas for each PHC clinic. Coupled with these KPIs are performance targets to meet, which are a guiding tool for performance measurement of quality of healthcare services.^6^ Key performance indicators are ways to measure how well the PHC clinics, or projects within PHC clinics, are performing in relation to the strategic goals and objectives.^7^ They provide important performance information that enables the NMs to understand whether they are on track towards the stated objectives and assist them in ensuring that PHC plans are focused on intended results.

Nurse managers (NMs) are responsible for leading the process of developing and implementing work plans that outline day-to-day activities for the PHC clinics that are aimed at facilitating achievement of the set target for each KPI.^8,9^ The District Health Information System facilitates analysis of data from PHC clinics and generation of reports on each KPI. Nurse managers are expected to retrieve these reports, analyse and interpret them to determine how targets have been met and to inform future work plans. The work plans assist managers to assign tasks, manage workflow and track the various components and milestones deadlines and to articulate strategies to employees in a way that improves team member focus and drive. Therefore, NMs should be highly creative in their thinking, and they should be able to piece together multiple pieces of seemingly unrelated information and test out new approaches in the pursuit of new meaning in support of patient nursing care.^3,10,11^

Several previous iLembe district annual reports reflected underperformance for various KPIs, thus making underperformance a constant problem in this district.^6^ Failure to achieve targets for KPIs appears to be a universal problem in South Africa as several other provinces have, in the same District Health Barometer report (2017–2018), been shown to be failing to meet the set targets for a number of KPIs.^2^ One example is couple-years of protection rate, which was reported to be below the national target of 60% at the national, provincial and district levels. The report showed that, the couple-years of protection rate was 48.2% at the national level, and by province it ranged from 35.1% in North West to 58.6% in the Western Cape, with North West, the Northern Cape and KwaZulu-Natal showing decreases of 7.6%, 6.6% and 5.7% points, respectively, from the previous year report.^6^ The rate varied widely in the districts, ranging from 35.8% to 85.2%, with only three districts in the country reaching the target of 60%.^2^

There is research evidence that poor planning is often responsible for failure to achieve goals and targets.^12^ Therefore, failure to achieve KPIs is attributed to poor planning. The researchers observed that audit reports often reflected that the NMs did not use KPIs in developing work plans. Yet KPIs are the core elements for measuring performance and should be used to guide future performance.^13^

Individual-level knowledge management practices and the task knowledge outcomes model^14^ was used as a theoretical framework to guide the study. In this model, knowledge management practices consist of ‘knowledge creation’, ‘knowledge sharing’ and ‘knowledge application’. Using the three concepts of the model (‘knowledge creation’, ‘knowledge sharing’ and ‘knowledge application’) guided the researchers in determining the practices of NMs in developing work plans, challenges that they experienced in this regard and possible strategies that could facilitate the use of KPIs when NMs are developing work plans for PHC clinics. The researchers considered that strategies facilitating the use of KPIs by NMs when developing work plans for PHC clinics could influence knowledge management practices and could be conceptualised as conceptual, contextual and operational knowledge gained through knowledge creation and knowledge sharing. This would subsequently result in knowledge application.

## Research aim and objectives

The aim of the study was to explore the experiences of NMs regarding the use of KPIs in developing work plans. The objectives of the study were to describe the current practices of NMs in developing work plans; identify and describe the challenges, if any, experienced by NMs with the use of KPIs in developing work plans; and explore strategies that can facilitate the use of KPIs by NMs to develop work plans.

## Research methods and design

A qualitative research design using an exploratory descriptive approach was employed. In line with the description by Grove, Burns and Gray,^15^ the exploratory descriptive qualitative research approach assisted the researcher in gaining an understanding of NMs’ experiences in the use of KPIs in developing work plans for PHC clinics in order to find ways to address the constant underperformance for various KPIs, which was attributed to the NMs not using KPIs in developing work plans.^15^

### Setting

The study was conducted in one of the four local municipalities within the iLembe district in KwaZulu-Natal. This local municipality has a total of 14 healthcare clinics, comprising one district hospital, one community health centre, seven fixed and five mobile PHC clinics. The focus of the study was on the seven fixed PHC clinics. All other healthcare institutions were excluded, as operations in these were managed differently to the fixed PHC clinics.^16^ All seven PHC clinics in the selected local municipality were included in the study.

### Study population and sampling strategy

The participants were the NMs from the fixed PHC clinics. All NMs except those who were reliefs while the NM was on leave were targeted. A purposive sampling method was used to select NMs who were either acting or permanently appointed as NMs. There were 28 NMs in the selected PHC clinics at the time of the study (four in each PHC clinic), out of which 20 met the inclusion criteria and were all sampled. Eight NMs were excluded based on the exclusion criterion, which was being a relief while the NM was on leave.

### Sample size

A minimum of one and a maximum of four NMs per PHC met the inclusion criteria and were all included in the study. In total, 20 NMs were included in the study, which was a census of all NMs who met the inclusion criteria.

### Data collection

All data were collected by one researcher who was working as a monitoring and evaluation manager in another local municipality within iLembe in KwaZulu-Natal, different to the one where the study was conducted. One-on-one semistructured interviews that were guided by an interview guide were used to collect data. Face-to-face interviews were conducted in a suitable venue, which was either at the PHC clinic or the subdistrict office, depending on the participants’ choice. The researcher ensured that the venue preferred was a private room with no distractions, such as people moving, noise or ringing telephones, and that the room was comfortable, appropriately ventilated with good light and with comfortable seating arrangements. The interview guide was prepared by the researcher in English and organised around a set of open-ended questions which assisted to narrow the interview to specific aspects of the phenomenon being studied while remaining open to how the participants respond.^15^ The interview guide was developed based on the research objectives and the theoretical framework that guided the study. The interview guide consisted of three broad questions in line with the three study objectives. Relevant probing questions based on the concepts of the theoretical framework (which are knowledge creation, knowledge sharing, and knowledge application) were listed under each broad question. The researcher, with previous experience and skills as a monitoring and evaluation manager, was able to conduct and control the semistructured interviews. The semistructured nature of the interviews allowed the participants to discuss their experiences in a manner that was comfortable to them and to discuss only the information that they were free and comfortable to discuss. This ensured that the participants did not feel pressured and that they were relaxed during the interviews. No interviewee was coerced to answer any specific question or to provide information than she felt uncomfortable sharing with the interviewer. That the researcher was from another local municipality and had no personal relationship with the study participants facilitated a free discussion during the interviews.

The interview sessions were scheduled on a first-come, first-served basis. However, the researcher ensured that she rotated scheduling interview sessions from one PHC to the next to ensure that all PHC clinics were included.

Before commencement of the interview, participants were screened for possible coronavirus disease 2019 (COVID-19) infection or exposure. The screening questions included whether the participant had recently been diagnosed with COVID-19 or had signs and symptoms of possible COVID-19 infection such as cough, sneezing, sore throat, and so on, or had recently been in contact with persons infected with COVID-19. The plan was to either cancel or postpone the interview sessions with all persons confirmed or suspected to be infected until it was safe. No such persons were identified during data collection. The COVID-19 preventative measures that applied during the interview sessions included the use of hand sanitiser, wearing of masks throughout the interview session (both supplied by the researcher) and the seating arrangement ensuring a distance of 1 m between the interviewer and the interviewee, who were seated squarely to avoid breathing onto each other’s faces.

Data saturation was reached after 16 participants were interviewed. Four additional interviews were conducted to confirm data saturation, thus giving a total of 20 NMs interviewed and a census of NMs who met the inclusion criteria. Nine NMs were overall in-charge for the PHC clinics and 11 were NMs for selected sections within the PHC clinic (acute illness, chronic illness and mother-and-child sections).

### Data analysis

Data analysis was done concurrently with data collection, preferably on the same day as the interview or within three days but ideally before the next interview. The reason for this was to ensure that each interview’s information was analysed as soon as possible while the session was still fresh in the mind of the interviewer. This facilitated comprehension and interpretation. Data were analysed using content analysis guided by Tesch’s open coding approach.^17^ Firstly, the recorded information was read and reread several times until it was fully understood, and thereafter it was read against the field notes, which assisted the researcher to get a clearer understanding of the information, and subsequently transcribed into a written format and compared and consolidated with the field notes. Again, the transcribed information was read, reread and compared with the original to ensure no meaning was lost during this process. A list of all topics was compiled, similar topics clustered together and preliminarily organised as major topics, unique topics and leftover topics. Related topics were grouped together, and an emerging list of categories was compiled. The preliminary analysis of data was accomplished by assembling data that belonged to each category, from which themes and subthemes emerged. Finally, existing themes and subthemes were identified and grouped together. A total of 11 themes and several subthemes were identified.

### Ethical considerations

Ethical clearance to conduct this study was obtained from the Institutional Review Board of Durban University of Technology (IREC 102/20). Gatekeeper permission was granted by relevant managers from the KwaZulu-Natal Provincial Department of Health, which included the research unit manager, the iLembe district manager, the PHC services general manager and NMs in charge of the seven PHC clinics in the selected local municipality.

## Results

The findings on demographic characteristics of the study participants are presented in Table 1.

**TABLE 1:** Demographic characteristics of the study participants.

Demographic variable	*n*	%
**Gender**		
Female	15	75
Male	5	25
**Age group**		
< 25 years	0	0
25–35 years	3	15
> 35 years	17	85
**Ethnicity**		
Black people	17	85
Indian people	3	15
Mixed race people	0	0
White people	0	0
**Experience as a nurse**		
< 5 years	0	0
5–10 years	6	30
> 10 years	14	70
**Experience as a nurse manager**		
< 2 years	6	30
2–10 years	9	45
> 10 years	5	25

Eleven themes and several subthemes emerged from the study. Because the majority of the themes and subthemes were interrelated, they were grouped together and collapsed into three major themes in line with the three study objectives. The three major themes included the practices of NMs in developing work plans, the challenges experienced by NMs in the use of KPIs in developing work plans and the strategies that, according to the NMs, could facilitate the use of KPIs in developing work plans. Table 2 presents the themes and subthemes and how they were collapsed into the three major themes.

**TABLE 2:** Themes and subthemes and how they were collapsed into the three major themes.

Major theme	Themes	Subthemes
1. Practices of NMs in developing work plans	1.1 Work plan not meeting the expected standard	Not using or incorrectly using KPIs in preparing work plans Reasons for work plan not meeting the expected standard
	1.2 Perception of nurse managers	Workload of NMs Not necessary to use KPIs in developing work plan
	1.3 Monitoring and evaluation strategies not facilitating the use of KPIs in developing work plan	Ever-changing monitoring and evaluation guidelines Feedback to nurse managers
2. Challenges experienced by NMs with the use of KPIs in developing work plans	2.1 Knowledge of NMs regarding their expected role and function	Knowledge related to developing work plans Knowledge related to interpreting KPIs and targets
	2.2 Training and support	Induction In-service education Support, mentoring and coaching
	2.3 Resource allocation	Computers and software to view DIS and other reports Time Human resources
	2.4 Exclusion of NMs from selected activities	Planning meetings Decision-making
3. Strategies that can facilitate the use of KPIs by NMs to develop work plans	3.1 Support in management role	Management support Peer support Subordinates
	3.2 Skills development and orientation	Induction In-service training Training curriculum
	3.3 Provision of resources	Computers and software Time Human resources
	3.4 Participative management	Involvement in decision-making Feedback

NMs, nurse managers; KPIs, key performance indicators; DIS, District Information System.

### Major theme 1: Practices of nurse managers in developing work plans

In healthcare services, including PHC clinics, NMs are responsible for planning, organising and directing health services in their departments to ensure that the goals and objectives are achieved consistently and that the services provided to the community are of the highest quality and standard.^10^ Under this role, one of the NMs’ core functions is to play a leading role in developing work plans that outline day-to-day activities for the PHC clinics which are aimed to facilitate achievement of the national targets.^3^ The plans are reviewed for accuracy and relevance by senior managers, who are first-line managers for PHC NMs. The participants attested that, as rated by senior management, the work plans prepared by the NMs were not meeting the expected standard because the NMs were either not using, or incorrectly using, the KPIs when preparing work plans. However, the participants gave various reasons for why their work plans were not meeting the expected standard. The reasons shared by the participants included that the KPIs were not written in a straightforward language that was easy for them to understand, which made interpretation and understanding difficult, especially in the absence of guidelines to follow when developing work plans and/or frequent changing of monitoring and evaluation guidelines. Another reason was poor or delayed feedback to NMs regarding submitted work plans, which made it difficult to learn and fully understand this process:

‘My major concern is interpretation of the KPIs. You will think you understand them, yet you are completely off the point; they are not written in simple language that is easy to understand.’ (PHC Clinic A, NM2)

However, some participants stated that the majority of NMs were reluctant to use the KPIs when preparing the work plan because they perceived this as unnecessary and just another senior management strategy to increase the workload for NMs:

‘Truly speaking, I am not even convinced why they are forcing us to these KPIs in developing work plans; it is just another strategy to increase our workload.’ (PHC Clinic F, NM1)

### Major theme 2: Challenges experienced by nurse managers with the use of key performance indicators in developing work plans

The management role of a NM in a PHC setting is to ensure that the resources required to execute daily activities are available, safe and fairly distributed. Nurse managers often experience a number of challenges in leadership roles that affect the effectiveness of their leadership and the quality of primary PHC services rendered.^18^ Similarly, the current study identified four challenges experienced by NMs with the use of KPIs in developing work plans. These included the knowledge of NMs regarding their expected role and function, training and support, resource allocation and exclusion of NMs from selected activities. The majority of participants stated that they were getting insufficient attention from the Department of Health concerning the provision of knowledge information related to developing work plans and interpreting KPIs and targets. The participants also stated that they never received induction training, in-service education and support or mentoring and coaching on data management when promoted to be NMs:

‘I must be honest; the task of using the KPIs in developing work plans is not an easy one, especially because most of us were never taught how to do this. Most of us were never inducted; you are just thrown into the deep end and you are expected to deliver.’ (PHC Clinic E, NM2)

However, they commended their peers for support, mentoring and coaching:

‘What has kept us going is having each other as NMs. We are always there for each other; we support and mentor each other.’ (PHC Clinic F, NM3)

As contributory factors in their inability to use KPIs in developing work plans, almost all study participants flagged the shortage of resources such as computers and software to view the District Information System and other reports, knowledge and skills to operate the computers, time and human resources:

‘One huge problem is the network. The clinic where I am working the network is most of the time down. Therefore, rather than being late in submitting my reports and the work plan, I use whatever little information I have to compile and submit these.’ (PHC Clinic F, NM2)

Furthermore, the participants indicated that excluding them from selected activities such as platforms where decisions were made and staff development forums such as workshops and in-service education training sessions affected their ability to use the KPIs in developing work plans:

‘As NMs, we work like machines; decisions are made for us. This is very frustrating and demotivating.’ (PHC Clinic C, NM1)

### Major theme 3: Strategies that can facilitate the use of key performance indicators by nurse managers in developing work plans

There is global recognition that competent managers are essential for ensuring that priority health needs are met, quality health services are delivered, and that resources are used effectively.^19^ However, there are a number of challenges experienced by NMs in the execution of their duties. Therefore, addressing the challenges identified in the work experiences of PHC NMs would go a long way in ensuring the successful implementation of health sector reforms and other nursing management responsibilities, including the use of KPIs in developing work plans.^19^ According to the study participants, the four strategies that can facilitate the use of KPIs by NMs in developing work plans include support in the management role, induction and in-service education, provision of resources and participative management.

Receiving support from selected people in the organisation such as management, peers and subordinates could facilitate the NMs’ use of KPIs in developing work plans:

‘… If senior managers can standardise and make regular their visits to PHC clinics for support and guidance.’ (PHC Clinic A, NM1)

Induction, in-service education and including KPIs and development of work plans in the training curriculum for the post-basic nursing management course were strategies that, according to the participants, could improve NMs’ knowledge and skill in the appropriate use of KPIs when developing work plans:

‘We cannot avoid frequent changing of KPIs and their targets due to ever-changing disease patterns, but it would be better that in-service trainings are provided every time the new KPIs are introduced and provision of ongoing support thereafter.’ (PHC Clinic C, NM1)

Equitable provision and allocation of resources such as computers and related software, Internet access, time and human resources for all PHC clinics, irrespective of location, whether urban or rural, was also listed among strategies to facilitate the use of KPIs in developing work plans:

‘Unlike the urban clinics that are well resourced, in our sub district we have huge resources constraints with one clinic having just one or two computers, no printer, and no fax machine.’ (PHC Clinic F, NM3)

The participants also highlighted participative management as another important strategy that in their opinion could facilitate the use of KPIs in developing work plans. All NMs should be involved in decision-making pertaining to the operations in the PHC clinics, including revision of current and deciding on new KPIs and targets, and should be given timeous composite feedback regarding PHC clinic operations:

‘It would be wise to invite NMs in meetings for KPIs revisions, as we are the ones who are at an operational level and who understands community dynamics.’ (PHC Clinic D, NM1)

## Discussion

The discussion focuses on the three objectives of the study, which were to describe the current practices of NMs in developing work plans; identify and describe the challenges, if any, experienced by NMs with the use of KPIs in developing work plans; and to explore strategies that can facilitate the use of KPIs by NMs to develop work plans. The three objectives incidentally correspond to the three major themes that emerged as the interrelated themes and subthemes were collapsed.

### Practices of nurse managers in developing work plans

#### Quality of submitted work plans

The work plans submitted by the NMs were reported to be of poor quality. Nurse managers’ inadequate knowledge regarding how to use the KPIs in developing work plans was reported as one of the major reasons responsible for this. Nurse managers should have capabilities to retrieve, analyse and interpret reports on clinic performance from the District Information System in order to be able to use KPIs effectively in preparing work plans.^3^ A number of participants acknowledged the inability of NMs to develop appropriate work plans and attributed this to a number of challenges experienced by the NMs. Other participants claimed that they were developing good work plans except that they were not using the KPIs, instead relying on their observation and knowledge regarding what the plans should entail, because in their opinion this was a more appropriate approach than using the KPIs. The action of the latter group is supported by Jeseviciute-Ufartiene, who stated that in the action of planning, managers often use their pure and rational thinking, reflection, experience of management and awareness.^20^ In addition, a number of the participants stated that the instruction to use KPIs in developing work plans was another senior management strategy to increase the NMs’ workload, which, according to them, was already high and heavy. This notion is supported by Syed et al., where they stated that the major problem in the efforts made by organisations to create conditions to achieve optimal production by the employees is that the interests of the organisation and the employees are most often not in the same direction. While the employees wish to have less work, the managers try to gain optimal production from existing workers by overloading them.^21^

#### Monitoring and evaluation strategies

Monitoring and evaluation strategies used in the PHC clinics were reported as not facilitating the use of KPIs in developing work plans because of the ever-changing monitoring and evaluation guidelines and inappropriate and sometimes delayed feedback given to NMs. The problem of ever-changing guidelines was compounded by delays in communicating changes to NMs, which left them misinformed regarding new developments. Monitoring and evaluation aims to provide managers, decision-makers and other stakeholders with regular feedback on progress in implementation, results and early indicators of problems to be corrected through reporting on actual performance against what was planned or expected.^4^ Thus, data sources from the district health management information system and other sources such as surveillance, population surveys and management information are integrated in the National Health Information Repository and Data Warehouse and made available to NMs for future planning.^22^

South Africa uses the National Department of Health Data Dictionary as a guideline to support the district health management information system policy. The Data Dictionary provides a reference point for selected health information standards to support healthcare activities and the most up-to-date version of data set specifications, particularly data elements, indicators and data validation rules and the organisational unit hierarchies.^8^ Guidelines are considered one of the most influential and effective tools for the promotion of evidence-based practice and a cure for the tension between healthcare cost and quality. They offer a chance to improve the quality of care by reducing practice variation and producing adherence to standards of good care.^23^ Implementing guidelines in healthcare systems often requires a multipronged approach that includes targeted intervention strategies with both clinicians and the healthcare system.^24^ The district health office is responsible for ensuring that all district health management information system users within the district (including subdistricts and clinics) have access to implementation guidelines.^25^

Poor and delayed feedback regarding PHC performance and submitted reports was another practice that negatively influenced the use of KPIs in developing work plans. Several researchers look at feedback together with audit and regard these as strategies for improving performance and supporting quality and safety in healthcare systems.^26,27^ Audit and feedback are widely used as a strategy to improve professional practice, either on its own or as a component of multifaceted quality improvement interventions based on the belief that healthcare professionals are prompted to modify their practice when given performance feedback showing that their clinical practice is inconsistent with a desirable target.^27^

#### Perceptions of nurse managers

This study identified that the perception of NMs influenced their use of KPIs in developing work plans. Self-efficacy is one’s belief in one’s ability to succeed in specific situations.^28^ An individual’s decision to adopt a given behaviour is a function of their intention to adopt that behaviour, which in turn is influenced by the three main drivers: attitudes towards the behaviour; subjective and descriptive norms; and perceived behavioural control.^29^

A number of NMs had negative perceptions about the use of KPIs in developing work plans. In their opinion, it was not necessary that the department be prescriptive that they should use KPIs in developing the work plans. According to the participants, an NM as the custodian of the work plan should be allowed liberty on how best to prepare the plan. Attitudes towards the behaviour, subjective and descriptive norms and perceived behavioural control are in part a function of an individual’s characteristics and past experiences.^30^ They are informed by beliefs about the positive and negative aspects of the behaviour and usually have an indirect effect on behaviour. Development of appropriate work plans could be enhanced by extracting managers’ perceptions and intuitions during the planning process.^20^

### Challenges experienced by nurse managers with the use of key performance indicators in developing work plans

Four key challenges identified as impeding the use of KPI in developing work plans included the knowledge of NMs regarding their expected role and function, training and support, resource allocation and exclusion of NMs from selected activities.

#### Knowledge of nurse managers regarding their expected role and function

The use of KPIs in developing work plans requires that NMs are able to interpret the KPIs. The current study identified that NMs lacked the knowledge regarding how to interpret the KPIs, which resulted in them developing substandard work plans. As alluded to by Muhammed, Doll and Deng, knowledge creation drives the behavioural processes related to knowledge management, such as knowledge application, which in turn impacts the individuals’ task-related knowledge.^14^ Regardless of the professional and managerial ability of an individual who is working within an organisation, if that certain individual does not have the right or the freedom to decide on certain management situations, performance cannot be attained within the organisation.^31^

#### Training and support

All persons involved in health information are required to have the ability to appropriately collect, consistently define, accurately aggregate, link, relate to knowledge and machine process health data accurately.^32^ However, in the current study, the NMs lacked this ability. The current study identified an absence of training opportunities and support as part of the challenges that were experienced by NMs, which resulted in their inability to use KPIs in developing work plans for their respective PHC facilities. The critical areas mentioned in this regard were induction, in-service education and training and support, mentoring and coaching.

Although under normal circumstances, NMs are supposed to have undergone a nursing management training course to prepare them for the management role, a number of NMs were promoted based on experience as a professional nurse without going through a formal course.^33^

Training is an act that plays a positive role in the success of the organisation that increases the skills and knowledge of the employees for the required purpose or task and allows the employees to obtain new skills and knowledge and become more effective and productive for the organisation.^34^ Effective leadership is the result of the appropriateness or fit between particular behaviours (being well trained and willing) and particular situations (being in an appropriate and welcoming context) to the functional necessities of each leadership organisation.^35^

It is also compulsory that employees assuming new positions are subjected to a full induction programme.^36^ A number of participants stated that no induction is provided for newly appointed NMs. Others stated that the induction sessions often do not address the critical challenges that NMs face in their role. Induction training is one of the forms of training that should be routinely conducted by organisations for its new, transferred, recategorised and promoted employees to help them settle quickly in their new roles.^34^

Furthermore, in order to keep staff members abreast with practice and new development, staff members in the healthcare establishment are subjected to a series of workshops and in-service education sessions coordinated at various operational levels, such as national, provincial or district levels. The participants in the current study stated that NMs are often not invited to the in-service training sessions related to data management, or when invited, they are unable to attend because they are too busy in the clinics. In-service training is an important undertaking in improving the performance of employees, simply because it has several positive effects on performance, which include self-development and gaining of new skills that enable employees to perform their tasks better with timely completion of tasks, all of which have a direct contribution towards better performance.^37^

Participants also mentioned that they did not get adequate support from their senior managers. When an individual is engaged in knowledge creation, this knowledge is typically applied by the individual in his or her work if he or she has all the resources necessary to put the new idea into practice.^14^

#### Resource allocation

Data sources from the District Health Information System and other sources such as surveillance, population surveys and management information are integrated in the National Health Information Repository and Data Warehouse and are made available to managers for future planning.^38^ Managers require access to computers and relevant software to be able to view these. Limited and disproportionate allocation of resources, including computers and software to view the District Health Information System and other reports, was another key finding of the current study. Some participants highlighted that they sometimes had to use their personal resources like computers and external drives to save reports from mother facility computers for them to have data available within their workplace for easy reference. One of the foremost challenges that healthcare systems are facing is the scarcity of resources in combination with rising demand for services, putting their sustainability in danger.^39^ Amid multiple demands and inadequate resources, there are times when nurses find it impossible to fulfil all nursing care requirements or choose not to complete all aspects of care for a variety of reasons by abbreviating the care, delaying the care or simply omitting the care.^39^ Missed care (nursing care left undone) is often caused by overwhelming demands on the nursing resource in specific contexts.^40^

The shortage of human resources has resulted in NMs spending most of their time attending to patients instead of focusing on management duties. The provision of health services is largely dependent on the sufficiency of the health workforce in terms of numbers, the quality of skills they possess, how and where they are deployed and how they are managed.^41^ The shortage of human resources in healthcare has been an ongoing problem in South Africa, particularly in rural areas where the unbalanced distribution between urban and rural areas leaves South Africa’s rural dwellers with 12% of its nurses.^24^ The 2016 report by the World Health Organization revealed that 57 countries, most of them in Africa and Asia, faced a severe health workforce crisis.^38^ Human Resources for Health South Africa recognises that the extensive and changing burden of disease in South Africa has several implications for human resource development and planning and states that ensuring an appropriate, trained and sustainable workforce is a priority for the South African health sector.^42^

#### Exclusion of nurse managers in selected activities

This study identified that NMs are often excluded from selected activities which are meant to be part of their role, such as planning and decision-making. Thus, the participants in the current study stated that their exclusion resulted in missed opportunities to make their input and voices heard on these platforms. Managers are persons who are formally appointed to positions of authority in an organisation and decision-making, which is part of problem-solving and problem analysis, which is an inherent activity of managers, allowing them to make decisions within and among the different management functions such as planning, organising, directing, controlling and staffing – all of which are brought to life and connected by decision-making.^43^

### Strategies that could facilitate the use of key performance indicators by nurse managers in developing work plans

The researcher sought the opinions of the study participants regarding strategies that could facilitate the use of KPIs by NMs to develop work plans. The proposed revision of the curriculum for post-basic training for nurses, induction of newly appointed NMs on data management, involvement of NMs in decision-making, management support to NMs and equitable distribution of resources in health facilities, irrespective of their geographical location, are the key strategies that could facilitate the use of KPIs by NMs to develop work plans.

#### Revision of curriculum for post basic training for nurses

Nurse managers are required to possess specific knowledge to be able to perform their role expectation, and knowledge is gained through education and training or experience in the field.^44^ Information acquired from school forms the basis of knowledge that a person can build up from, and it is usually the one that prunes an individual.^45^ However, most participants stated that they never received any training on data management neither from their basic training as nurses nor during their post basic nurse training. Nevertheless, the minimum educational requirement for NMs varies from one employer to another, with some employers allowing an experienced registered nurse to assume the role of NM without completing a nursing management course.^46^

#### Induction of newly appointed nurse managers

Despite the importance of the role of NMs, a number of new NMs received little, if any, formal preparation, yet they were expected to lead the operations in their units, including development of work plans.^31^ Thus, even though there is a data dictionary that is provided to the clinics’ personnel to define the indicators and data elements, sometimes NMs see the information as being too complex and not easy to understand, especially for those NMs who had not been trained in data management. Participants recommended that the Department of Health should either introduce a new system on the induction and orientation of newly appointed NMs on KPIs or strengthen the existing one. Kunene agrees strongly with the personal growth of NMs, saying that if the health professionals are inducted effectively, they will have the motivation and enthusiasm to implement the departmental goals and priorities related to service delivery in their workstations.^47^

#### Involvement of nurse managers in decision making

In South Africa, the healthcare system remains centralised, with narrow decision space over most matters such as finance and human resources, policies, etc., for managers from district level downwards.^46^ This results in little or no involvement of NMs in decision-making. The majority of participants stated that they were not involved in the process of developing KPIs for the programmes that they were expected to manage; instead, data management personnel were prioritised. They proposed that the department should include NMs in the process of developing KPIs because they were the ones playing leading roles in the process of developing work plans. In the context of changing healthcare goals, delivery approaches and health management, health managers and leaders with adaptable and relevant capabilities are critical to high-quality systems of healthcare delivery and thus should be involved in decision-making.^46^

#### Management support for nurse managers

Lack of support is a significant work-related problem that NMs are often exposed to. This results in NMs who are unproductive, discontented and more vulnerable to burnout and depersonalisation.^48^ Participants showed dissatisfaction with the level of support they were receiving from senior management. Knowledge is a process and does not exist independent of human action.^49^ Yet organisations often face difficulties, including lack of senior management commitment, when applying knowledge management systems.^50^

#### Equitable distribution of resources in health facilities

The study participants recommended that equal allocation of all resources in PHC facilities, including computers and network systems, would assist PHC facilities to access their data timeously for planning. In order to provide quality services, a number of basic resources need to be available, regardless of how well-utilised the service is.^51^ The participants also proposed that the department should establish and strengthen the system of facilities to assess one another so as to create opportunity for them to learn best practices from one another. By comparing one facility’s operations to those of other organisations, there is the potential to learn and improve performance, because benchmarking one’s business operations with defined metrics helps track progress and reach goals faster.^52^

### Limitations

This study was carried out on a very small scale involving one small district with a limited number of participants, and thus the results are not generalisable. The involvement of senior management in the study and a retrospective review of work plans would have enriched the findings from the study.

### Recommendations

Recommendations are made in relation to service delivery, nursing education and training and further research to address the challenges experienced by NMs. These recommendations are aimed at the different tiers of management, including the ward or unit, line manager or senior manager and human resources levels.

Despite the importance of the role of NMs, a number of new NMs receive little, if any, formal preparation, yet they are expected to lead the operations in their units including development of work plans.^31^ Thus, it is recommended that human resources should ensure that there is formal induction of newly appointed NMs and that the induction programme cover relevant aspects of the expected role of NMs. The human resources division should also ensure adequate provision of human resources in PHC clinics to allow NMs to focus on their management role instead of carrying out tasks meant to be carried out by subordinates.

The line manager or senior manager should ensure that support is available for NMs in the form of in-service training, workshops on data management and ensuring that NMs are actively involved in decision-making. Training is an act that plays a positive role in the success of the organisation, increasing the skills and knowledge of the employees for the required purpose or task and allowing the employees to obtain new skills and knowledge and become more effective and productive for the organisation.^32^ They should also ensure adequate allocation of resources so that all NMs have relevant tools to practise and ensure that NMs are skilled in the use of available tools, such as computers. In order to provide a certain service, a number of basic resources need to be available, regardless of how well-utilised the service is.^53^

At the ward or unit level, NMs should support each other, identify mentors and benchmark good practices from each other to facilitate the execution of the use of KPIs in developing work plans and other management tasks. The dedicated guidance that is received through mentoring and coaching is more beneficial than formal teaching because they inspire, energise and facilitate learning and are deemed to be a highly effective way to help people, through talking and increasing self-direction, self-esteem, efficacy and accomplishments in the new millennium.^54,55,56^

In addition, a broader study involving more districts and provinces is recommended. The current study identified gaps in areas such as knowledge of NMs regarding their role, support received by NMs and resource allocation to PHC clinics located in rural areas. These are critical areas requiring further research, as they negatively impact the performances of NMs and therefore service delivery and management.

## Conclusion

Relevant knowledge management practices are essential for the development of task knowledge (NMs’ knowledge regarding the use of KPIs in developing work plans) to ensure that tasks are appropriately executed. Although several other challenges such as inadequate resources allocation impacted the NMs’ use of KPIs in developing work plans, this study identified a knowledge gap which could be filled through training, in-service education, induction, mentoring and support as the major cause of this problem. Therefore, as alluded by Muhammed, Doll and Deng, knowledge creation and knowledge sharing can facilitate knowledge application and improve the practice of NMs in the use of KPIs in developing work plans.
